# Case Report: A Case of Hereditary Gingival Fibromatosis With a High Level of Human β Defensins in Gingival Epithelium

**DOI:** 10.3389/fimmu.2021.763026

**Published:** 2021-10-29

**Authors:** Ge Gao, Qing Tian, Anpeng Han, Rongxia Yang, Fan Shi, Dong Chen

**Affiliations:** Department of Stomatology, The First Affiliated Hospital of Zhengzhou University, Zhengzhou, China

**Keywords:** non-syndromic hereditary gingival fibromatosis, clinical manifestation, human β-defensins, case report, oral defense

## Abstract

Hereditary gingival fibromatosis [HGF, (MIM 135300)], a rare benign oral condition, has several adverse consequences such as aesthetic changes, malocclusion, speech impediments, and abnormal dentition. However, relatively few studies have addressed the beneficial effects of thick gingival tissues in resisting external stimuli. In this report, we present a unique case of a family affected by HGF that manifests as a ‘healthy’ gingiva. Human β-defensins (hBDs) are known to play a pivotal role in the clearance and killing of various microbes, and contribute to maintaining a healthy oral environment, which is currently emerging research area. However, the expression pattern and localisation of hBDs in patients with HGF have not yet been reported. hBD-2 and hBD-3 in the pedigree we collected had relatively elevated expression. High hBD levels in the gingival tissue of patients from the family may be beneficial in protecting oral tissue from external stimuli and promoting periodontal regeneration, but their role and the mechanisms underlying HGF need to be clarified.

## Introduction

HGF is a rare benign oral condition that can present as generalised slow thickening and non-bleeding fibrous enlargement of keratinized gingivae (1). This condition may lead to aesthetic changes, malocclusion, speech impediments, and abnormal dentition (2). However, to the best of our knowledge, none of the studies on patients with HGF reported the beneficial protective effects of thick gingival tissues against external stimuli. Our study describes a unique case of patients in a family affected by HGF, with relatively elevated serum hBD levels, pink and firmly consistent gingivae, alveolar bone thickening to various degrees, and a higher regrowth rate of gingival tissues.

The gingival epithelium is constantly exposed to varying microbial environments and physical and chemical stimuli generated by mastication and ingestion. The function of the epithelium in the host response has been increasingly recognised to serve to form a rigid mechanical barrier against periodontopathogenic bacteria. However, it also protects oral tissue by sensing and initiating the innate immune response, including secretion of various pattern recognition receptors and antimicrobial host defence peptides (3). Antimicrobial peptides are significant multifunctional modules in the natural defence system (3). Human β-defensin-2 and -3 (hBD2 and hBD3) are two representative microbial peptides that are the members of the defensin family.

Considering the relatively healthy oral condition of these patients, we speculated that certain genic mutations might serve beneficial for protecting the host from dental plaque when a series of toxic factors are produced by bacteria penetrating their thick epithelial wall, and the generally thickened gingival tissue associated with this pedigree may be a desirable consequence of the battle between antimicrobial peptides and bacterial invasion. Further studies are warranted to elucidate the unique gingival features. Therefore, the difference in the expression of hBD-2 and hBD-3 between HGF and normal gingival epithelium was chosen as the target of this study to investigate the chemical barrier provided by hBDs.

## Case Presentation

**Figure 1:**
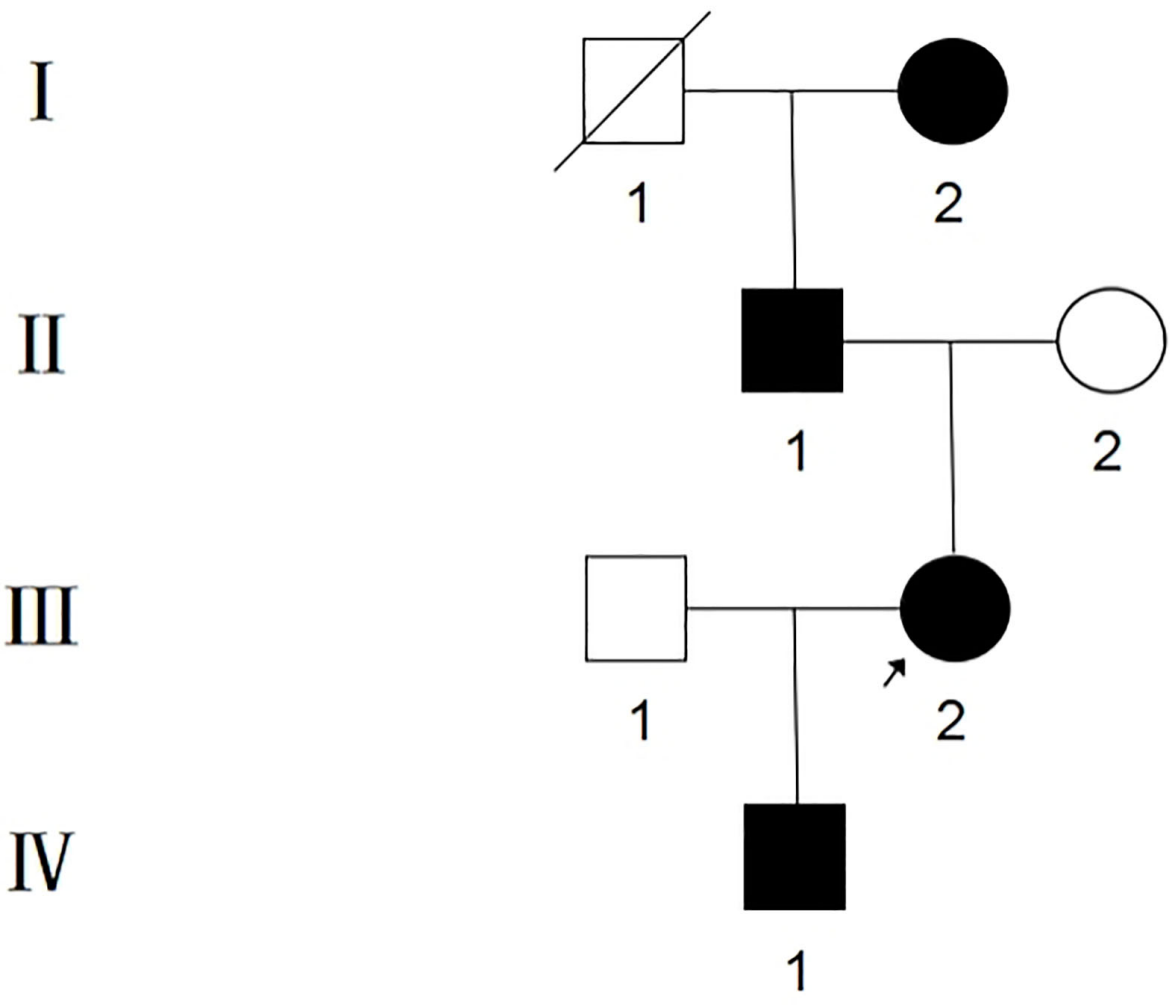
Pedigree of Chinese family with NHGF. Affected individuals are indicated by blackened symbols. Circles and squares denote female and male members respectively, and a slash through a symbol denotes a deceased individual. The proband is indicated by the arrow.

**Figure 2:**
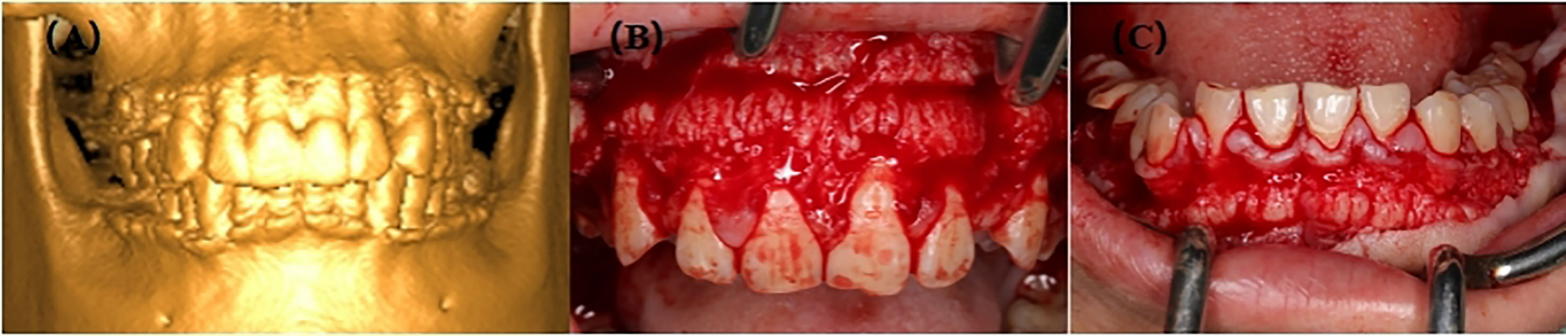
**(A)** 3D image of III-2; **(B)** Preoperative picture of maxilla from III-2; **(C)** Preoperative picture of mandible from III-2.

**Figure 3:**
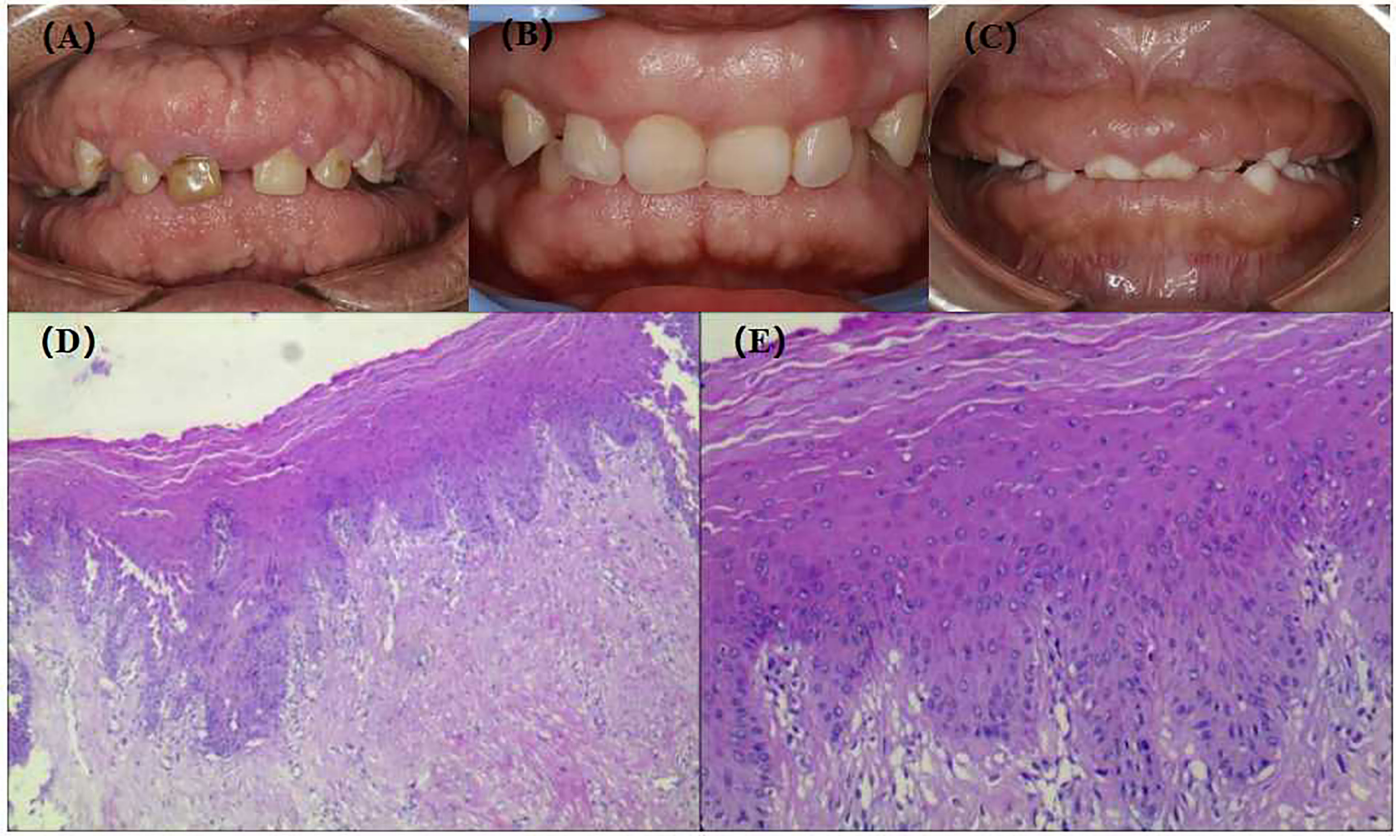
Extra-oral images, intra-oral images and HE staining of gingival tissue of patients with NHGF. **(A)** II-1, 63 years old; **(B)** III-2, 35 years old; **(C)** IV-1, 5 years old; **(D)** HE staining of gingival tissue III-2(40×); **(E)** HE staining of gingival tissue III-2(200×).

The proband, III-2, female, 34 years old, complained about gingival hyperplasia affected appearance and eating in the Stomatological Hospital of Zhengzhou University. His father and son had similar symptoms. Gingivectomy was performed at the ages of 15, 18, 20, and 25 because of gingival hyperplasia. All individuals were classified as affected or unaffected based on clinical features, family history, and histopathological manifestations, as described by Hart et al. (**Figure 1**). NHGF, which affects both sexes, is inherited through autosomal dominance. The family members with NHGF manifested a relatively ‘healthy’ gingiva, and their periodontal biotype was classified as a thick flat type on clinical examination (2, 4). Patients among the family members generally had fair oral hygiene, and even those who were first-generation presented firmly consistent gingivae, which consisted of dense fibrous connective tissue that felt tough and tuberculous on palpation. Furthermore, we found that the alveolar bone of these patients was significantly thickened during the gingivectomy (**Figure 2**). The first-generation I-2 aged 91 years showed relatively fair oral hygiene and had pink enlarged gingiva with marked and abundant stippling covering almost the entire crown. II-1, the father of the proband aged 63 years, exhibited significant hyperplasia of the posterior dental area and had teeth that were partially or entirely engulfed by fibrotic tissue (**Figure 3A**). Marked, abundant stippling was observed in the intraoral image of the proband (**Figure 3B**). IV-1, son of the proband, who was 5 years old, exhibited extensive hyperplasia of the entire gingival margin, papilla, and attached gingiva of both the maxillary and mandibular dentition (**Figure 3C**). A strikingly distinctive facial appearance was also observed in this pedigree, comprising both hypertrichosis as previously reported and abnormal changes in the nose and mouth. All affected individuals manifested a coarsened facial appearance, marked nasal and upper lip protrusion, and an open bite. They had flat nasal bridges, broad noses, and bow mouths. Their hair and eyebrows were bushy, whereas their weights and heights were all within the normal range and did not manifest any intellectual disability.

The gingival tissue HE staining of the patients in the pedigree was characterised by hypocellular and hypovascular dense fibrous connective tissue covered by an integrated stratified squamous epithelium. In addition, prominent deposition of collagen fibres underlying the gingival epithelium with acanthosis and extended long slender rete ridges were observed (**Figures 3D, E**), which is consistent with the histological characteristics of gingival fibromatosis.

**Figure 4:**
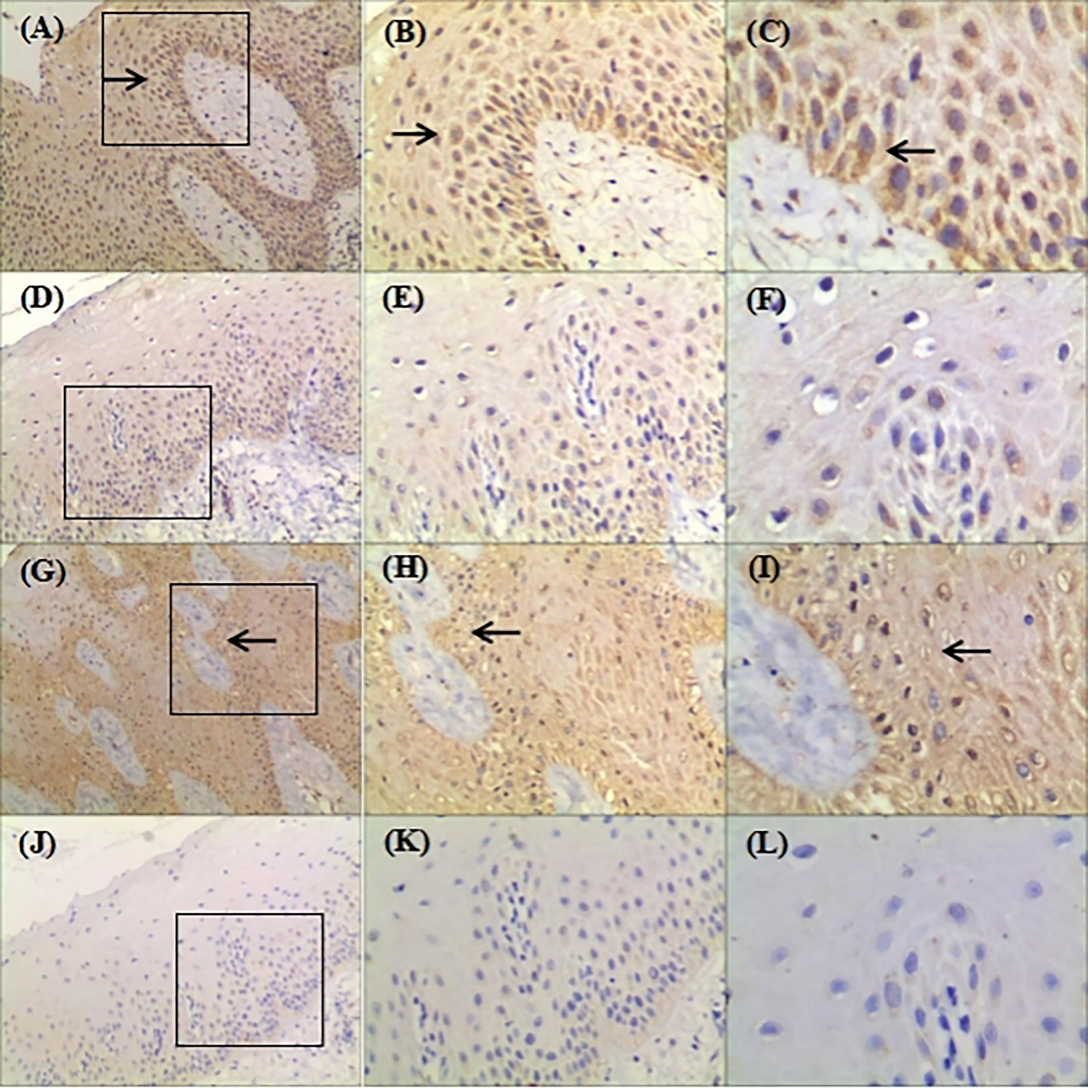
Representative images showing expression of hBD-2 and hBD-3 in gingival tissue samples from the family (original magnification ×100, ×200, ×400). **(A–F)**: Gingival samples were stained with rabbit polyclonal IgG antibody to hBD-2 (1:300) using standard IHC protocol; Circled areas in **(A)** was amplified in **(B)** and **(C)**; **(D)** was amplified in **(E)** and **(F)**; **(A–C)** were derived from patient with NHGF; **(D–F)** were derived from a healthy control gingival biopsy; **(C)** hBD-2 was detected in the cytoplasm; Arrows indicate positive staining. **(G–L)**: Gingival samples were stained with rabbit polyclonal IgG antibody to hBD-3 (1:400) using standard IHC protocol; Circled areas in **(G)** was amplified in **(H, I)**; **(J)** was amplified in **(K, L)**; **(G–I)** were derived from patient with NHGF; **(J–L)** were derived from a healthy control gingival biopsy; **(I)** hBD-3 was detected in the nuclei and cytoplasm. Arrows indicate positive staining.

The immunohistochemistry showed that hBD-2 was mainly confined to the cytoplasm, whereas hBD-3 was detected in the cell nuclei and cytoplasm (**Figures 4C, I** arrows). In addition, hBD-2 staining was intense, brownish-red, in the cytoplasm of tissue sections from the NHGF group (**Figures 4A–C**). In contrast, weak staining was observed in the control group sections, which were slightly yellow (**Figures 4D–F**). The hBD-3 staining was significant, brown, in both the nuclei and cytoplasm of sections from the NHGF group, whereas those of the control group were blue (**Figures 4G–L**). Furthermore, hBD-2 and hBD-3 were generally expressed in all epithelial layers of the NHGF group sections but were not detected in the underlying connective tissue layer. In addition to the corneum, granulosum, and spinosum, basal cells were clearly stained, and the expression density gradually increased from the corneum to the basal layer. The strongly stained areas were distributed in bands or sheets, indicating that hBD-2 and hBD-3 positively expressed in the basal layer of the gingival epithelium tissue from the pedigree.

We treated the patients with basic periodontal treatment, full-mouth alveolar bone repair, and gingivoplasty. The wound was irradiated by laser healing mode for 15 min after the operation, and periodontal maintenance treatment lasted throughout the treatment cycle. The gingival tissue condition of the patients was greatly improved, the dentition was neat, the occlusion was stable, the shape was beautiful, and the patients’ self-confidence was greatly improved.

## Discussion

HGF usually presents as an isolated incidence of gingival hyperplasia or occasionally appears as an oral manifestation of certain syndromes. This article describes the unique clinical characteristics of NHGF, which is a rare benign oral condition characterised by a progressive increase in keratinized gingiva showing an autosomal dominant inheritance or, infrequently, an autosomal recessive inheritance (5). Presently, the clinical diagnosis of NHGF is still based on histopathological examination. Although highly recurrent characteristic hyperplastic gingiva can cause severe functional and aesthetic problems, the gingival tissue from the pedigree we collected might show better resistance to detrimental external stimuli. Recently, a series of innate host defence molecules, such as antimicrobial host defence peptides in the human gingiva, have emerged as a research focus, and have proven their indispensable roles in maintaining periodontal health. Antimicrobial host defence peptides contribute to establish a state of ‘controlled’ immuno-inflammatory surveillance by depolarizing and disrupting microbial cell membrane integrity (6). Because of their polymorphous nature and paramount significance in the natural host defence, these molecules have been termed ‘host defence peptides’. The first antimicrobial peptides identified in the oral epithelial tissue were hBDs, which show prominent specific activity against various microbes, and have recently attracted considerable attention (3). These peptides stimulate local dendritic cells and recruit T-cells into the nearby gingival epithelium to link innate and acquired immunological responses (7, 8).

The current research focus has shifted from antimicrobial capabilities to effects on the immunoregulation of congenital and acquired immune responses (9). For example, defensins interact with numerous inflammatory factors, regulate epithelial cell proliferation, participate in periodontal regeneration, promote wound healing, induce or curb pro-inflammatory cytokines, facilitate or inhibit angiogenesis, enhance chemokine production, promote chemotaxis of diverse leukocytes, mediate degranulation of mast cells, and regulate the host cell gene expression (10–13). More specifically, hBD-2 activates the antigen presentation activity of dendritic cells and stimulates the production of interferon (IFN)-γ, tumour necrosis factor (TNF)-α, interleukin (IL)-1β, IL-6, and IL-22 production. Furthermore, it serves as a surveillance factor by inhibiting IL-17 production *via* suppression of cytokine signalling 3 (SOCS3) (3). In addition to being a chemotactic factor for macrophages, monocytes, and mast cells, hBD-3 is a chemokine for immature dendritic cells and CD45 RA+/CD4+ T lymphocytes, which reside in the oral mucosa and play essential roles in immunity. With the exception of periodontal tissue inflammation and injury, pro-inflammatory cytokines such as IL-1β, TNF-α, IL-6, and IL-17 can modulate systemic diseases (14–16). hBD-3 effectively inhibits TNF-α and IL-6 accumulation to induce potential anti-inflammatory properties, leading to inflammation resolution (10, 17). With increasing available information about the beneficial effects of hBDs in humans, it is important to study the functions of these defensins in periodontal immunoregulation and fibrous tissue regeneration in more detail. hBD-3 can be used to improve root surface biocompatibility and promote periodontal ligament fibroblast attachment and proliferation (11). Coincidentally, we observed that the regrowth rate of the gingival tissues from patients with NHGF pedigree was higher than that of normal gingiva after gingivectomy, which is consistent with the results of previous investigations. Moreover, hBD-3 can interact with the host defence and inflammation mechanisms in tissue reconstruction in articular cartilage. Specifically, it participates in remodelling articular cartilage tissue by increasing the secretion of cartilage-degrading matrix metalloproteinases and reducing the production of endogenous regulatory factors, such as tissue inhibiting factors of metalloproteinases 1 and 2 (18). hBDs may facilitate tissue regeneration, which is important for the recovery from gingival recession or periodontal surgery, further investigations should aim to determine the beneficial effects of hBD-associated NHGF. Provided that it proves to be beneficial to the physiological development of periodontal tissue, we will continue to monitor this family to explore the potential key role of NHGF in periodontal immunoregulation and fibrous tissue regeneration.

Previous research on the biochemical pathogenesis of NHGF was primarily focused on connective tissue cells of patients with NHGF, while ignoring the pathological features of the epithelium and the interaction between gingival epithelia and underlying fibroblasts (19–21). hBD-2 and hBD-3 were mainly distributed in the basal layer of the pedigree investigated in our study (**Figures 4A, B, G, H** arrows). The basal layer is an interfacial surface with the lower lamina propria, which contains blood vessels and contributes to the formation of the gingival epithelium by providing nutrients and potentially impacts the recognition of signals from the body. Although junctional epithelium frequently causes inflammation, in contrast to the basal layer, hBDs have not been detected (22). The cells of the junctional epithelium are comparatively undifferentiated, which indicates that the expression of hBDs in the oral stratified squamous epithelium depends on the normal differentiation of epithelial cells (23). Specific interactions between the gingival epithelium and the underlying lamina propria may drive the development of HGF.

In the HGF family we studied, the expression of hBDs in gingival epithelium was statistically higher, and their oral health status was relatively healthy, suggesting that hBDs may be beneficial in regulating host responses to oral pathogen challenges to maintain homeostasis of the oral environment. We could not determine the relationship between elevated expression of hBDs and gingival thickening at present because of the insufficient number of samples. We speculate that hBD-2 and hBD-3 could be potential facilitators of communication between the gingival epithelium and underlying lamina propria without compromising the host.

## Patient’s Perspective

These patients did not have any intellectual disability or other systemic diseases, presenting only a clinically NHGF phenotype. They plan to continue gingival cosmetic repair to achieve greater confidence.

## Data Availability Statement

The datasets used and/or analyzed during the current study are available from the corresponding author on reasonable request.

## Ethics Statement

The studies involving human participants were reviewed and approved by the Medical Ethics Committee of the First Affiliated Hospital of Zhengzhou University, China (2021-KY-0039). Written informed consent to participate in this study was provided by the participants’ legal guardian/next of kin. Written informed consent was obtained from the individual(s), and minor(s)’ legal guardian/next of kin, for the publication of any potentially identifiable images or data included in this article.

## Author Contributions

DC conceived the idea of the study and the experimental design. GG performed the immunohistochemistry, image analysis, and data analysis, and contributed to the preparation of the manuscript. QT and AH contributed to the conception and design of the experiments and contributed reagents and analysis tools. RY and FS prepared the figures and tables. All authors reviewed the paper.

## Funding

The National Natural Science Foundation of China (82170920); the Medical Science and Technology Research Project of Henan Province (22170124).

## Conflict of Interest

The authors declare that the research was conducted in the absence of any commercial or financial relationships that could be construed as a potential conflict of interest.

## Publisher’s Note

All claims expressed in this article are solely those of the authors and do not necessarily represent those of their affiliated organizations, or those of the publisher, the editors and the reviewers. Any product that may be evaluated in this article, or claim that may be made by its manufacturer, is not guaranteed or endorsed by the publisher.

## Abbreviations

HGF, Hereditary gingival fibromatosis; NHGF, Non-syndromic Hereditary gingival fibromatosis; hBDs, human β- defensins; hBD-2, human β-defensin-2; hBD-3, human β-defensin-3.

## References

[1] Häkkinen L Csiszar A. Hereditary Gingival Fibromatosis: Characteristics and Novel Putative Pathogenic Mechanisms. J Dent Res (2007) 86:25–34. doi: 10.1177/154405910708600104 17189459

[2] Gawron K Lazarz-Bartyzel K Potempa J Chomyszyn-Gajewska M. Gingival Fibromatosis: Clinical, Molecular and Therapeutic Issues. Orphanet J Rare Dis (2016) 11:9. doi: 10.1186/s13023-016-0395-1 26818898 PMC4729029

[3] Lijian J. An Update on Innate Defense Molecules of Human Gingiva. Periodontol 2000 (2011) 56(1):125–42. doi: 10.1111/j.1600-0757.2010.00364.x 21501240

[4] Shao Y Yin L Gu J Wang D Lu W Sun Y. Assessment of Periodontal Biotype in a Young Chinese Population Using Different Measurement Methods. Sci Rep (2018) 8(1):11212. doi: 10.1038/s41598-018-29542-z 30046153 PMC6060136

[5] He L Ping FY. Gingival Fibromatosis With Multiple Unusual Findings: Report of a Rare Case. Int J Oral Sci (2012) 4(4):221–5. doi: 10.1038/ijos.2012.53 PMC3633067 22955199

[6] Premratanachai P Joly S Johnson GK McCray PB Jr Jia HP Guthmiller JM. Expression and Regulation of Novel Human Beta-Defensins in Gingival Keratinocytes. Oral Microbiol Immunol (2004) 19:111–7. doi: 10.1111/j.0902-0055.2002.00127.x 14871351

[7] Greer A Zenobia C Darveau RP. Defensins and LL-37: A Review of Function in the Gingival Epithelium. Periodontol 2000 (2013) 63(1):67–79. doi: 10.1111/prd.12028 23931055 PMC3744237

[8] Gursoy UK Pollanen M Kononen E Uitto VJ. A Novel Organotypic Dento-Epithelial Culture Model: Effect of Fusobacterium Nucleatum Biofilm on B-Defensin-2, -3, and LL-37 Expression. J Periodontol (2012) 83(2):242–7. doi: 10.1902/jop.2011.110177 21692631

[9] Li L Jiang H Chen R Zhou J Xiao Y Zhang Y. Human Beta-Defensin 3 Gene Modification Promotes the Osteogenic Differentiation of Human Periodontal Ligament Cells and Bone Repair in Periodontitis. Int J Oral Sci (2020) 12(1):13. doi: 10.1038/s41368-020-0078-6 32350241 PMC7190824

[10] McCormick TS Weinberg A. Epithelial Cell-Derived Antimicrobial Peptides Are Multifunctional Agents That Bridge Innate and Adaptive Immunity. Periodontol 2000 (2010) 54(1):195–206. doi: 10.1111/j.1600-0757.2010.00373.x 20712640 PMC3816379

[11] Wang H Watanabe H Ogita M Ichinose S Izumi Y. Effect of Human Beta-Defensin-3 on the Proliferation of Fibroblasts on Periodontally Involved Root Surfaces. Peptides (2011) 32(5):888–94. doi: 10.1016/j.peptides.2011.02.002 21320561

[12] Gursoy UK Kononen E. Understanding the Roles of Gingival Beta-Defensins. J Oral Microbiol (2012) 4:15127. doi: 10.3402/jom.v4i0.15127 PMC3290911 22389759

[13] Chotjumlong P Khongkhunthian S Ongchai S Reutrakul V Krisanaprakornkit S. Human β-Defensin-3 Up-Regulates Cyclooxygenase-2 Expression and Prostaglandin E2synthesis in Human Gingival Fibroblasts. J Periodontal Res (2010) 45(4):464–70. doi: 10.1111/j.1600-0765.2009.01259.x 20337883

[14] Finetti M Omenetti A Federici S Caorsi R Gattorno M. Chronic Infantile Neurological Cutaneous and Articular (CINCA) Syndrome: A Review. Orphanet J Rare Dis (2016) 11(1):167. doi: 10.1186/s13023-016-0542-8 27927236 PMC5142346

[15] Awad F Georgin-Lavialle S Brignier A Derrieux C Aouba A Stankovic-Stojanovic K. Chronic Myelomonocytic Leukemia as a Cause of Fatal Uncontrolled Inflammation in Familial Mediterranean Fever. Orphanet J Rare Dis (2015) 10:76. doi: 10.1186/s13023-015-0295-9 26076658 PMC4485869

[16] Wei A Ma H Zhang L Li Z Guan Y Zhang Q. Clinical Analysis of Chronic Active EBV Infection With Coronary Artery Dilatation and a Matched Case-Control Study. Orphanet J Rare Dis (2021) 16(1):50. doi: 10.1186/s13023-021-01689-5 33509232 PMC7845094

[17] Semple F Webb S Li HN Patel HB Perretti M Jackson IJ. Human Beta-Defensin 3 has Immunosuppressive Activity *In Vitro* and *In Vivo* . Eur J Immunol (2010) 40(4):1073–8. doi: 10.1002/eji.200940041 PMC2948537 20104491

[18] Varoga D Pufe T Harder J Schroder JM Mentlein R Meyer-Hoffert U. Human Beta-Defensin 3 Mediates Tissue Remodeling Processes in Articular Cartilage by Increasing Levels of Metalloproteinases and Reducing Levels of Their Endogenous Inhibitors. Arthritis Rheum (2005) 52(6):1736–45. doi: 10.1002/art.21090 15934078

[19] Ye X Shi L Cheng Y Peng Q Huang S Liu J. A Novel Locus for Autosomal Dominant Hereditary Gingival Fibromatosis, GINGF3, Maps to Chromosome 2p22.3-P23.3. Clin Genet (2005) 68(3):239–44. doi: 10.1111/j.1399-0004.2005.00488.x 16098013

[20] Beer HD Gassmann MG Munz B Steiling H Engelhardt F Bleuel K. Expression and Function of Keratinocyte Growth Factor and Activin in Skin Morphogenesis and Cutaneous Wound Repair. J Investig Dermatol Symp Proc (2000) 5(1):34–9. doi: 10.1046/j.1087-0024.2000.00009.x 11147673

[21] Häkkinen L Koivisto L Gardner H Saarialho-Kere U Carroll JM Lakso M. Increased Expression of β6-Integrin in Skin Leads to Spontaneous Development of Chronic Wounds. Am J Pathol (2004) 164(1):229–42. doi: 10.1016/S0002-9440(10)63113-6 PMC1602209 14695336

[22] OuharaKKomatsuzawaHYamadaSShibaHFujiwaraTOharaM. Antimicrobial Peptides in the Oral Environment: Expression and Function in Health and Disease. J Antimicrobial Chemother (2005) 55(6):888–96. doi: 10.1093/jac/dki103 15886266

[23] Dale BA Kimball JR Krisanaprakornkit S Roberts F Robinovitch M O’Neal R. Localized Antimicrobial Peptide Expression in Human Gingiva. J Periodont Res (2001) 36(0022-3484):285–94. doi: 10.1034/j.1600-0765.2001.360503.x 11585115

