# Supplementary material for: Case Report: A Case of Hereditary Gingival Fibromatosis With a High Level of Human β Defensins in Gingival Epithelium
Source: Front Immunol. 2021 Oct 29;12:763026. doi: 10.3389/fimmu.2021.763026 (PMC8594454; doi:10.3389/fimmu.2021.763026)

Table 1. mean optical density of NHGF group and Control group.

|  | NHGF | Normal | Z | P |
| --- | --- | --- | --- | --- |
| hBD-2 | 0.17 (0.16–0.1975) | 0.12 (0.1125–0.13) | -4.655 | <0.001* |
| hBD-3 | 0.175 (0.1425–0.23) | 0.135 (0.12–0.1475) | -3.637 | <0.001* |

Note: * the difference was statistically significant, P<0.05


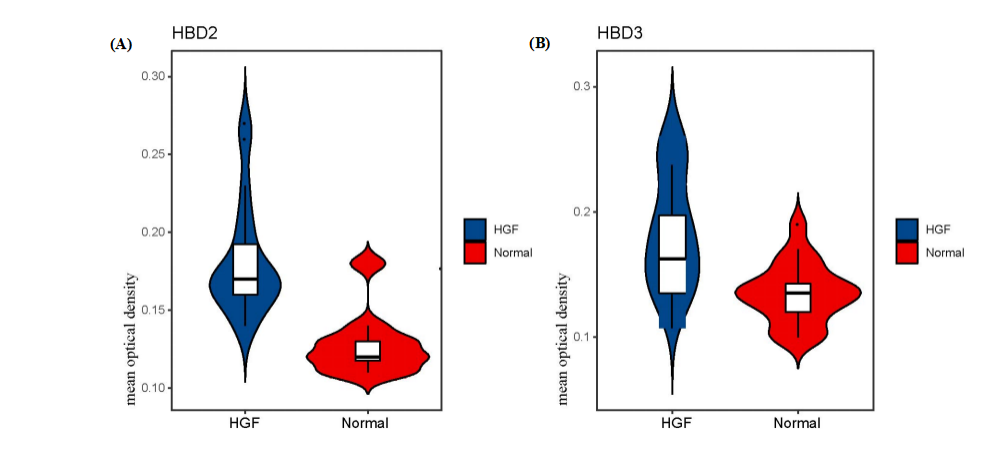

Supplement: Supplementary file 1 [file Table_1.docx]
